# Eradication of scrapie with selective breeding: are we nearly there?

**DOI:** 10.1186/1746-6148-6-24

**Published:** 2010-05-04

**Authors:** Marielle B Melchior, Jack J Windig, Thomas J Hagenaars, Alex Bossers, Aart Davidse, Fred G van Zijderveld

**Affiliations:** 1 Central Veterinary Institute of Wageningen UR, P.O. Box 65, 8200 AB Lelystad, the Netherlands; 2 Animal Breeding and Genomics Centre, Wageningen UR, Livestock Research, P.O Box 65, 8200 AB Lelystad, the Netherlands

## Abstract

**Background:**

Following EU decision 2003/100/EC Member States have recently implemented sheep breeding programmes to reduce the prevalence of sheep with TSE susceptible prion genotypes. The present paper investigates the progress of the breeding programme in the Netherlands. The PrP genotype frequencies were monitored through time using two sets of random samples: one set covers the years 2005 to 2008 and is taken from national surveillance programme; the other is taken from 168 random sheep farms in 2007. The data reveal that although the level of compliance to the breeding programme has been high, the frequency of susceptible genotypes varies substantially between farms. The 168 sheep farms are a subset of 689 farms participating in a postal survey inquiring about management and breeding strategies. This survey aimed to identify how much these strategies varied between farms, in order to inform assessment of the expected future progress towards eradication of classical scrapie.

**Results:**

On the one hand, we found that compliance to the national breeding program has been high, and the frequency of resistant genotypes is expected to increase further in the next few years. On the other hand, we observed a large variation in prevalence of the scrapie resistant PrP genotype ARR between farms, implicating a large variation of genetic resistance between farms. Substantial between-flock differences in management and breeding strategies were found in the postal survey, suggesting considerable variation in risk of scrapie transmission between farms.

**Conclusions:**

Our results show that although there has been a good progress in the breeding for scrapie resistance and the average farm-level scrapie susceptibility in the Netherlands has been significantly reduced, still a considerable proportion of farms contain high frequencies of susceptible genotypes in their sheep population. Since 2007 the breeding for genetic resistance is voluntarily again, and participation to selective breeding can decrease as a result of this. This, together with the patterns of direct and indirect contact between sheep farms, might present a challenge of the aim of scrapie eradication. Communication to sheep owners of the effect of the breeding programme thus far, and of the prospects for classical scrapie eradication in The Netherlands might be essential for obtaining useful levels of participation to the voluntary continuation of the breeding programme.

## Background

Classical scrapie is a transmissible spongiform encephalopathy (TSE) in sheep and goats occurring world-wide, from which the earliest described clinical cases date back hundreds of years. The hereditary component in scrapie was suspected for many years [1,2], and in the 1990s the association of susceptibility to this prion disease with the polymorphisms of the ovine PrP gene was elucidated [3-8]. Genetic resistance to classical scrapie is associated with polymorphisms at three sites on the PrP gene (i.e. at codons 136, 154 and 171). These polymorphisms combine to produce five different alleles of the PrP gene: "ARR", "ARQ", "ARH", "AHQ" and "VRQ"[3]. In most breeds, animals carrying the VRQ allele are at greatest risk [9] and if the VRQ allele is not present ARQ-homozygous animals are at greatest risk [10]. ARR-homozygous and ARR-heterozygous animals, with exception of ARR/VRQ animals, have a significantly reduced risk of developing scrapie as compared to animals of other genotypes [11-13]. This implies the possibility to select animals with scrapie resistant genotypes for breeding.

From 2002, due to the risk to human health posed by the potential presence of BSE in sheep, an increase of the number of scrapie surveillance samples to be genotyped was implemented by the EU. In 2003, as a result of EU decision 2003/100/EC, this was followed by setting up breeding programmes to reduce the incidence of susceptible PrP genotypes from that year onwards [14]. The aim of the EU decision was to improve sheep resistance to both scrapie and BSE, and this could be achieved by selecting for the same resistant ARR allele. The ideal result would be obtaining a major reduction of human exposure risk to both BSE and scrapie, as well as control of the animal health problem posed by scrapie in sheep [15].

In the Netherlands a more stringent breeding programme was implemented compared to most other EU countries in 2004. Here selection for scrapie resistance already started in the 1990s on a voluntary basis. Mainly the larger purebred sheep breeds took part in this breeding programme [16]. In 2004 all Dutch sheep flocks consisting of more than 10 ewes were obliged to use a ram with the ARR/ARR genotype, whereas EU decision 2003/100/EC [14] only required a breeding programme for purebred sheep flocks of high genetic merit, and this was still voluntary until 1 April 2005. In 2005 all Dutch sheep flocks were obliged to use rams with the ARR/ARR genotype. The feasibility of this programme was ensured by the early voluntary start of breeding for scrapie resistance in purebred sheep in the Netherlands, which provided for enough breeding rams with the ARR/ARR genotype in 2004 and 2005. The obligatory programme was in force until June 2007, after which selection for scrapie resistance became voluntary again.

The ultimate aim of a national breeding programme is eradication of scrapie through a reduction of the reproduction ratio of the disease (R_0_) below 1. This can be achieved with a ARR allele frequency below 100%, preventing loss of all variation in the PrP gene. The frequency at which R_0 _<1 occurs depends on the distribution of scrapie resistance over flocks, and on the contact structure between flocks and farms. A uneven distribution of the overall scrapie resistance over farms may result in some highly susceptible flocks, despite low overall levels of susceptible genotypes. In addition, if some farms have much more contacts with other sheep farms than others, they run a higher risk of acquiring scrapie infection and of infecting contact farms. These heterogeneities may promote scrapie persistence especially in the scenario that farms with high-contact-rate are relatively likely to also have low scrapie resistance. The aim of this paper is two-fold. The first objective is to assess variation in prevalence of the resistant PrP gene ARR and the compliance to the breeding programme at the farm level. For this purpose we used the genotyping results from the 168 farms sampled in 2007 (further indicated as the Genotyping Survey Farms or GSF). We compared the genotype frequencies of the GSF sample with an independent genotyping sample from the statutory active scrapie surveillance of healthy slaughtered sheep and fallen stock, which was taken between 2005 and 2008 (further indicated as the National Surveillance Sample or NSS). This enabled us to assess possible biases and to draw conclusions on the progress of selective breeding on a national level. The second objective is to obtain more insight in the differences in management, breeding strategies and contact profiles between sheep farms in the Netherlands, and for this a questionnaire was used (689 farms, 2007, further indicated as the Survey Farms, SF). The 168 GSF were part of the SF.

## Results

### Management, breeding strategies and contact profiles across farms

The average farm size for both the GSF and the SF farms was around 31 ewes per farm (see Table 1). This is somewhat lower than the average number of ewes on sheep farms in the Netherlands with more than 3 NGE (NGE is the Dutch equivalent of the European Size Units, ESU), which was approximately 40 in 2007 (Statistics Netherlands, http://www.cbs.nl). The difference between these average numbers express the fact that the Netherlands contains quite a large number of very small sheep flocks owned by non-professional sheep farmers.

**Table 1:** Results from the postal survey.

	GSF sample	SF sample
	(n = 168)*	**(n = 689)**

**Ewes per farm**		

Mean	31.7	31.1

St. dev	56.7	48.6

Presence of breeds**		

Texelaar	56.6%	55.8%

Swifter	29.8%	34.0%

Zwartbles	16.0%	12.5%

Bleu de Maine	8.9%	3.3%

Blauwe Texelaar	7.1%	3.9%

Minor breeds	30.4%	38.3%

**No. breeds per farm**		

1	53.4%	49.6%

2	34.5%	39.5%

3	10.1%	8.7%

> 3	2.0%	2.3%

ARR/ARR selection ram *		

All breeds	84.9%	68.3%

Texelaar	89.9%	71.2%

Swifter	91.5%	76.4%

Zwartbles	81.5%	58.1%

Bleu de Maine	85.7%	76.5%

Blauwe Texelaar	92.3%	85.0%

Minor breeds	72.9%	55.9%

* The 168 farms sampled for genotyping (GSF) were part of the postal survey.

** Because farms may contain animals of more than one breed (see No. breeds per farm), these percentages of breeds do not add up to 100%.

Survey results on farm sizes, breeds used and application of selective breeding. Farms in the genotyped group (GSF) are compared to the rest of the survey (SF minus GSF) as a reference group.

The majority of sheep from the GSF and the SF belong either to the Texel breed or to the related Swifter breed (originating from - Texel × Vlaming). More than 80% of sheep are Texel, Swifter or crossbreeds with these breeds (Table 1). Almost 50% of farms use more than one breed, and 12% use 3 or more breeds.

Almost 85% of the farms in the GSF, stated that they still selected a scrapie resistant ram for breeding, and this was somewhat higher compared to the rest of the survey i.e. SF minus GSF (68%, see Table 1).

The majority of sheep farmers buy one new breeding ram every year or every other year (70 - 80%), and less than 10% buy more than one ram per year, which is in good agreement with the distribution of flock sizes in the Netherlands, and the amount of ewes which can be covered with one ram. It is known that farmers with few sheep often borrow a ram for breeding, or bring their ewes to the ram a few weeks, therefore not buying a ram does not exclude ram-mediated contact with other farms. Less than 1% of flocks are not bred.

Only 20% of sheep farmers state that they purchase ewes on a yearly basis. The difference between the trading volumes of male and female animals is considered important, since trading females might present a greater risk as lambing is generally considered to be a significant event for transmission of scrapie infections [17-19].

Between 30 and 40% of sheep are grazing at other farms, however less than 10% comes into direct (nose to nose) or indirect (faecal) contact with other sheep. These outcomes are in line with the proportion of farms (7 - 9%) which receive sheep from other farms for grazing at their premises.

### PrP frequencies in samples from Genotyping Survey Farms, and the National Surveillance Sample

**Figure 1:**
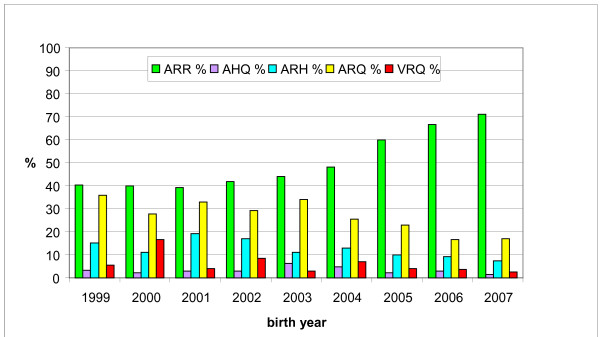
**Farm survey PrP allele frequencies**. Mean PrP allele frequencies of all sheep from the 168 farms sampled in this study divided by birth cohort.

The genotyping results from the GSF shows an increase in ARR allele frequency from around 40% for female sheep born in or before 2001, to more than 70% for females born in 2007 (see Figure 1). The increase in ARR frequency from 2002 can be assumed to be a result of voluntary selection, the larger increase for sheep born in 2005 is related to the implementation of the compulsory breeding programme. The average PrP allele frequencies in the GSF sample from 2007 are 58.4% ARR, 22.7% ARQ, 10.5% ARH, 3.2% AHQ and 4.4% VRQ (data not shown). The ARR allele frequency in the birth cohorts is increasing significantly (Chi-square test, P < 0.0001) from cohort -1999 to 2007.

The genotype frequencies in the 2006 and 2007 birth cohorts on each farm sample can be used to study the compliance with the compulsory use of scrapie resistant rams in 2005 and 2006. The use of these ARR/ARR rams will result in offspring with at least one ARR allele, the presence of offspring without ARR allele proves non-compliance. To quantify non-compliance we have therefore in Table 2 counted the number of animals without ARR allele in different birth cohorts (within the GSF sample), as well as, by birth cohort, the number of farms with at least one animal without ARR allel in that birth cohort. Here the birth cohorts up until 2003, before ram selection became compulsory, are grouped together. Based on the results for the 2006 and 2007 cohorts we conclude that the compliance in 2005 and 2006 was around 80%.

**Table 2:** Compliance to the breeding programme *.

	Birth cohorts in GSF sample				
	
	1999 - 2003	2004	2005	2006	2007
**Farms**					

Number (%)	95/128 (74.2)	54/110 (49.1)	40/127 (31.5)	21/121 (17.4)	21/145 (14.5)

**Animals**					

Number (%)	268/742 (36.1)	115/437 (26.3)	74/618 (12.0)	36/628 (6.1)	53/887 (6.0)

* The use of ARR/ARR rams in the Dutch breeding programme in GSF sample, compulsory in 2004 (for farms > 10 ewes) and 2005 (all farms).

Presence of animals without ARR allele in different birth cohorts in the GSF sample, measured in the number of farms with at least one animal without ARR allel in the given birth cohort(s), and measured in the number of animals without ARR allele in the given birth cohort(s) across all farms sampled.

**Figure 2:**
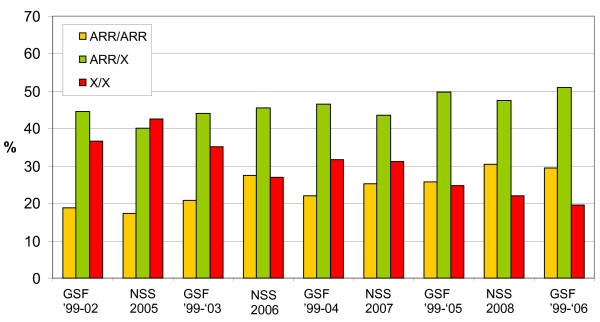
**Alignment of GSF and NSS genotyping samples**. The average ARR homozygote (ARR/ARR), heterozygote (ARR/X), and non-ARR (X/X) frequencies in the NSS sample, which only contains animals over 18 months of age, was aligned with the GSF sample by exclusion of the birth cohorts which are not present in the NSS sample. In detail: the NSS of 2008 (of animals over 18 months) does not contain animals from the 2007 birth cohort; the NSS of 2007 does not contain animals from the 2006 and 2007 birth cohorts; etc. GSF '99-02: genotype frequencies of birth cohorts 1999 - 2002; GSF '99-03: genotype frequencies of birth cohorts 1999 - 2003; etc. NSS 2005: genotype frequencies of the National Surveillance Sample from 2005; NSS 2006: genotype frequencies of the National Surveillance Sample from 2006; etc.

In the NSS sample, we find an increase in ARR allele frequency, from less than 38% in 2005 to 55% in 2008. The increase in ARR allele frequency in the NSS is lagging behind the farm sample due to the fact that the NSS sample consists of animals with higher average age, as the national surveillance contains slaughtered and fallen sheep over 18 month's of age. We are able to align the two samples by excluding the consecutive birth cohorts from the GSF sample which are not part of the NSS sample, and repeat this for all four years of the NSS sample (see Figure 2). In this way we are able to visualize that, although the samples are obviously from different sheep populations, the ARR allele prevalence is increasing in both populations, and at the same time the prevalence of sheep without ARR allele is decreasing.

### PrP frequencies across surveyed farms

**Figure 3:**
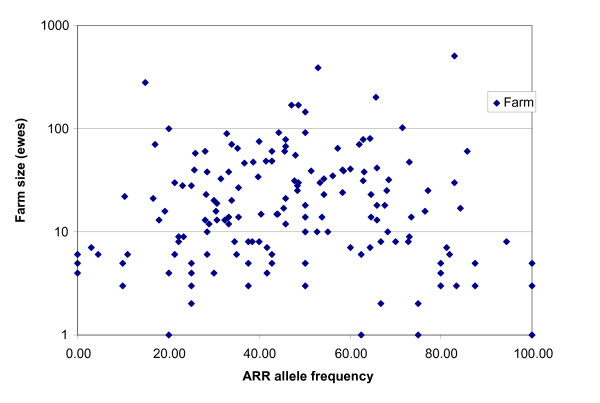
**Farm level PrP frequency variation**. Scatterplot of the farm size, measured by the number of ewes against ARR allele frequency measured on the farm (n = 168).

ARR allele frequencies varies between farms from 0 to 100% (Figure 3), while the overall frequency was 58%. The variability is largest in the farms with less than 10 ewes. In farms were more than 25 ewes were typed the ARR frequency varies from 18.6 to 76.2%. The observed heterozygosity varies from 25.7 to 88.6% in these farms, while the calculated expected heterozygosity ranges between 32.5 and 71.2%. Observed heterozygosities are always higher than expected, except for the farms with the lowest ARR frequency and lowest heterozygosity.

The excess of heterozygotes translates into a FIS value of -0.254, the most likely explanation being ongoing selection for ARR alleles. The differences between farms results in a FST value of 0.096, indicating that differences in allele frequencies between farms are larger than expected by chance.

## Discussion

Due to the strong linkage of PrP genotype at codons 136, 154 and 171 with the susceptibility to classical scrapie in sheep, classical scrapie is a unique infectious disease for which disease control may be reached relatively easy through selective breeding. Initially implementation of the EU decision 2003/100/EC [14] for resistant PrP genotypes was motivated by the potential presence of BSE in sheep. By now, better economic results from scrapie free flocks and better animal health as purposes of scrapie eradication are receiving more emphasis in discussions in the Netherlands.

Participation in the Dutch breeding programme which became compulsory in 2004, was made voluntary again in 2007. As a result, breeding with scrapie resistant ARR/ARR rams is now, although still recommended by sector organizations, not legally compulsory anymore for flocks which are not of high genetic merit. The results of the selection for scrapie resistance in the Netherlands are good with a significant decrease in scrapie incidence in the last four years [20]. However, only if a positive case of scrapie is found through the active surveillance system, which is still in place by EU regulations, a sheep farmer may face the financial consequences if genotyping results of his flock shows he has not implemented a breeding programme for scrapie resistance.

Our genotyping survey (GSF) aimed to measure the genotype frequency distribution of the resistant ARR allele in the population of non-studbook ewes, which produces 90% of lambs each year. In the broader general survey (SF) we inquired about management practices, and invited farmers to participate in the genotyping survey (GSF). Both the voluntary character of participation and the response rate imply that the results are not a random sample of the Dutch population, and conclusions should be drawn with caution.

The results of the survey show that (Table 1) in the group of the GSF sample, the use of scrapie resistant rams was higher than in the rest of the survey (SF minus GSF sample). If breeders acquire their rams without requiring a resistant genotype, the probability of acquiring a resistant genotype depends on the source of acquisition. If the ram is bought from a studbook breeder, from one of the five main breeds (see Table 1) the chance of purchasing a scrapie resistant ram is more than 95% (data not shown). However if the ram is bought from a non-studbook breeder, the chances of scrapie resistance are highly variable (Figure 3).

With the current ARR allele frequency in the Netherlands being just over 55% in the female sheep population, further progress towards more scrapie resistance will require the continued use of scrapie resistant rams for mating, especially in flocks of low resistance. To monitor the progress towards more scrapie resistance in the Dutch sheep, continued genotyping of a sample of the active surveillance seems to be an easy and reliable way. However, since we do not know the exact age of sheep genotyped in the NSS, besides being over 18 months, only substantial changes in the ARR allele frequencies will be noticed. In the current situation, with highest ARR allele frequencies in younger animals, and the mean ARR frequency around 50%, it will be difficult to detect possible reduction in the growth of the ARR allele frequency in the active surveillance samples in the next 2-3 years.

When aiming for eradication of scrapie, we should bear in mind that the effect of breeding for genetic resistance may need to be supported by a reduction in contacts between farms that may cause transmission. This may be of relevance especially given the current large variation in genetic resistance at the flock level in the Netherlands (Figure 3), which may lead to a protracted presence of a small number of flocks in which scrapie can still easily spread. The spread of scrapie from flock to flock is supported by direct contact, i.e. the purchase of infected animals, as well as by indirect contact, i.e. by grazing on infected premises, as can be learned from previous research [2,12,19,21,22]. The results from the postal survey on farm management show that nearly all farms regularly purchase rams, and only 20% of farms frequently purchase ewes. Given the high excretion of infectious material during lambing [12,19], the risk of acquiring a scrapie infection seems to be higher when a purchased animal is a ewe than when it is a ram. The risk of transmission of scrapie through grazing with other sheep flocks, as is practiced by less than 10% of flocks under Dutch circumstances is unclear. To understand the relative risk of different between-flock contact routes, further studies should be performed.

## Conclusions

The results from this study show that considerable progress has been made in breeding for scrapie resistance. However both the large variation in scrapie resistance between flocks, and the variation in the direct and indirect contacts between flocks complicate scrapie eradication. These complicating factors, together with the potential reduction in ARR ram selection after the legal obligation was removed in 2007, call for continued monitoring of the effects of selective breeding in the coming years. Communication to sheep owner of these effects, and of the prospects for scrapie eradication in The Netherlands might be essential to ensure good participation to the voluntary continuation of the breeding programme.

## Methods

### Postal survey amongst Dutch sheep farms

In 2007 a random sample of 6000 sheep farms, which were not part of the voluntary breeding programme (in which mainly studbook flocks take part) of the national Dutch Animal Health Service (GD, Gezondheidsdienst voor Dieren, Deventer) was selected for a postal survey. Management, breeding and contact with other farms were surveyed, and the possibility of PrP genotyping was offered. A total of 689 postal surveys were returned. The survey inquired about the size of the flock in terms of number of animals and hectares for grazing, housing and the management surrounding lambing. Although most farms do not participate in studbooks, the survey inquired on the breeds present on these farms. Furthermore, the contact with sheep from other farms through buying or selling of ewes or rams, through direct (nose to nose) or indirect (faecal) contact with neighbouring farms, or through grazing at other farms was investigated. The survey also inquired whether a scrapie resistant ram(s) was still used for breeding. This question was relevant because the survey was conducted after withdrawal of the rule for compulsory use of ARR/ARR rams for breeding in the Netherlands.

### PrP frequencies in samples from surveyed farms, and the national surveillance

From the 689 farms that completed the postal survey, 168 accepted the offer to genotype (part of) their animals. A maximum of 35 ewes were blood sampled per farm, and samples were taken proportionally per birth year cohort. If farmers owned less than 35 ewes, a maximum of 5 rams could be sampled too. Samples were sent to the Central Veterinary Institute (CVI, Lelystad) for analysis of the polymorphisms at the PrP gene codons 136, 154 and 171 through Taqman probe analysis. A total of 3314 sheep were genotyped, including 3207 ewes born between 1995 and 2007.

Frequencies of PrP alleles from the farm survey were compared with frequencies from a sample of the national active surveillance programme. The latter data consisted of random samples from both the healthy-slaughter and the fallen-stock streams. These samples were taken from 2005 - 2008 thus providing information on the temporal trend of the genotype frequencies at a national level [20].

### PrP frequencies across surveyed farms

Frequencies of PrP alleles and genotypes were compared across individual farms.

To test whether PrP alleles were randomly distributed across farms and whether PrP alleles were randomly distributed across individuals within farms Wrights F-statistics were estimated [23,24]. These F-statistics indicate whether more (or less) heterozygotes occur than expected under the Hardy Weinberg equilibrium. They are based on three measures of heterozygosity: HT is the expected heterozygosity based on the whole population, Hs is the mean of the expected heterozygosities in each subpopulation (in our case farm) and HI is the observed frequencies of heterozygotes (I = for individuals). FST summarizes whether allele frequencies diverge among subpopulations and is calculated by Ht-Hs/Ht. Values > 0 indicate that farms differ in the frequencies of PrP alleles, most likely because selection for scrapie resistance has been different across farms. The FIS statistics summarizes whether deviations within farms occur, and is calculated as Hs-Hi/Hs. If, for example selection is in operation, negative values are expected (i.e. excess of heterozygotes) if mating of relatives is practiced, positive values are expected (i.e. lack of heterozygotes). To avoid influence of sampling bias, only allele frequencies of farms with more than 25 genotyped animals were used for analysis.

## Authors' contributions

MM participated in the design of the study and carried out the survey including the analysis and interpretation of the data and drafted the manuscript. JW participated in the design of the study and the interpretation of the data and calculated the breeding statistics. TH participated in the design of the study and the interpretation of the data. AB participated in design of the study and supervised the genotyping study. AD participated in the collection of samples and genotyping. FZ participated in the design of the study and the interpretation of the data. All authors read and approved the final manuscript.
